# Predictors of Long-Time Survivors in Nonmetastatic Colorectal Signet Ring Cell Carcinoma: A Large Population-Based Study

**DOI:** 10.1155/2022/5393571

**Published:** 2022-08-17

**Authors:** Wuguang Zhang, Wenqian Gong, Changhai Wu, Mengting Li, Xiaolong Tu

**Affiliations:** ^1^Oncology Department of Integrated Traditional Chinese and Western Medicine, The Affiliated People's Hospital of Ningbo University, Ningbo 315100, China; ^2^Traditional Chinese Medicine Department, The Affiliated People's Hospital of Ningbo University, Ningbo 315100, China; ^3^Department of Acupuncture, Ningbo Medical Center Lihuili Hospital, Ningbo 315040, China; ^4^Department of Gastroenterology, The Affiliated People's Hospital of Ningbo University, Ningbo 315100, China; ^5^Department of Oncology, Ningbo Hospital of Traditional Chinese Medicine, China

## Abstract

**Background:**

Colorectal signet ring cell carcinoma (SRCC) is a rare and distinct subtype of colorectal cancer (CRC), with extremely poor prognosis and aggressive tumor biological behavior. In this study, we aimed to analyze the clinicopathological characteristics and to identify the independent predictors of long-time survivors (LTSs) of nonmetastatic colorectal SRCC.

**Methods:**

Patients diagnosed with nonmetastatic colorectal SRCC were extracted from the Surveillance, Epidemiology, and End Results (SEER) database. We compared and analyzed the clinicopathological characteristics between LTSs (patients survived over 5 years) and non-LTSs (patients survived of or less than 5 years). Afterwards, multivariate logistic regression analysis was used to identify independent predictors of LTSs, which were further used to construct a nomogram model to predict the probability of being LTSs.

**Results:**

We enrolled 2050 patients with nonmetastatic colorectal SRCC, consisting of 1441 non-LTSs and 609 LTSs. Multivariate logistic regression analysis revealed that race, marital status, tumor infiltration, lymph node involvement, and primary tumor treatment were independent predictors of LTSs. In addition, these five parameters were incorporated into a nomogram model to predict the probability of being LTSs. In terms of the model performance, the calibration curve revealed good agreement between observed and predicted probability of LTSs, and receiving operator characteristic curve showed acceptable discriminative capacity in the training and validation cohorts.

**Conclusion:**

Collectively, we analyzed and profiled the clinicopathological characteristics of LTSs in patients with nonmetastatic colorectal SRCC. Race, marital status, T stage, N stage, and primary tumor treatment were independent predictors of LTSs.

## 1. Introduction

Colorectal cancer (CRC) remains the second most common cause of cancer-associated mortality [1], posing great challenge to human health. Most CRCs are differentiated adenocarcinomas, followed in order by mucinous adenocarcinomas, signet ring cell carcinomas (SRCC), and squamous cell carcinomas [2]. The description of SRCC was first proposed in 1951 by Laufman and Saphir [3], which is gradually characterized by predominant intracytoplasmic mucin production in tumor cells (>50% size of cell), with the unique appearance of a signet ring [4, 5]. Primary colorectal SRCC is a rare and unique entity of CRC, accounting for approximately 1% of all CRC [6]. Despite the low incidence, accumulative attention has been paid to colorectal SRCC due to its rarity, aggressiveness, and distinct biological properties.

According to relevant studies, colorectal SRCC mostly originates from undifferentiated stem cells of colorectal mucosa, which might be the intrinsic cause for the high proportion of poor differentiation/undifferentiation, rapid tumor growth, diffuse infiltration, massive lymphatic involvement, great risk of distant metastasis, and peritoneal metastasis [7, 8]. In terms of demographic factors, SRCC is more commonly seen in young populations and female patients [9].

Several studies have investigated the prognostic factors for SRCC, showing that age, sex, tumor grade, tumor size, and primary tumor site are independently associated with patient survival [10]. The prognosis of patients with colorectal SRCC is dismal, with a 5-year overall survival (OS) rate of 25% [11], which is far lower than that of colorectal adenocarcinoma. The treatment of colorectal SRCC has been improved in recent years. Surgical resection remains the mainstay for resectable colorectal SRCC. Other therapeutic approaches, including chemotherapy, radiation, and targeted therapy, have also been widely applied to improve patient prognosis. The advanced clinical management of colorectal SRCC has improved patient prognosis. However, due to the rarity of colorectal SRCC, few studies have investigated the specific characteristics of patients with colorectal SRCC who survive for a long time, which hinders the survival improvement in colorectal SRCC.

To this end, we extracted eligible patients with colorectal SRCC from the Surveillance, Epidemiology, and End Results (SEER) database to retrospectively analyze the clinicopathological characteristics and predictors of long-time survivors (LTSs). For better clinical application, we further constructed an easy-to-use nomogram model to predict LTSs in nonmetastatic colorectal SRCC, followed by the assessment of model performance.

## 2. Materials and Methods

### 2.1. Data Source and Patient Selection

The SEER database is an authoritative source of data for cancer incidence and patient survival by including population-based data from 18 registration centers and covering approximately 30% of the US population [12]. SEER∗Stat software (version 8.3.6, released on August 8, 2019) was used to select qualified patients with nonmetastatic colorectal SRCC from 2004 to 2015. Since data from the SEER database are publicly available and deidentified, no institutional review or informed consent from patients was required in this study.

**Figure 1:**
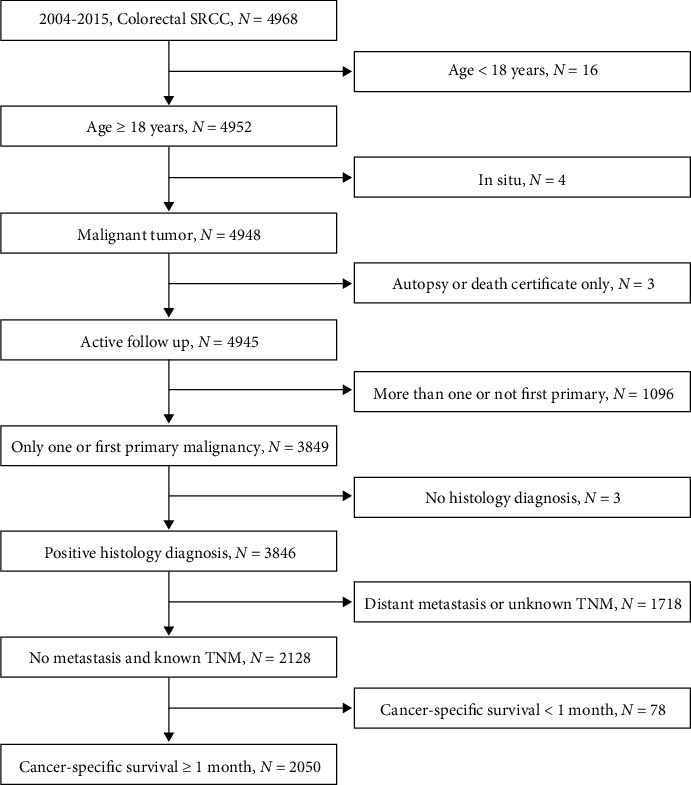
Flow chart of patient selection based on inclusion and exclusion criteria.

Patients included in the present study should meet the following criteria: (1) patients histologically diagnosed with colorectal SRCC between 2004 and 2015 based on the International Classification of Diseases in Oncology (ICD-O-3) (ICD-O-3: 8490); (2) patients aged 18 years or more; (3) patients were subjected to active follow-up whose cancer-specific survival (CSS) was no less than 1 month; (4) colorectal SRCC should be the only or first primary malignancy; and (5) patients without distant metastasis and (6) TNM stage should be available. Based on the above-described inclusion and exclusion criteria, 2050 eligible patients were finally included in our study (Figure 1).

### 2.2. Variables and Outcomes

In this study, patients were divided into two groups according to their survival time. LTSs referred to patients whose CSS was longer than 5 years, while patients had CSS no more than 5 years were defined as non-long-time survivors (NLTSs). CSS was defined as the duration from initial tumor diagnosis to death caused by colorectal SRCC.

The baseline characteristics of patients were extracted from the SEER database for analysis. Age at diagnosis was categorized into four groups, namely, ≤40, 41-55, 56-70, and>70 years. Race was recorded as black, white, and other (mainly including American Indian, Asian, and Pacific Islander). Sex was recorded as male and female. Marital status included married and unmarried, and the latter included single, divorced, separated, and widowed. Tumor grade was recorded as well-differentiated/moderately differentiated and poorly differentiated/undifferentiated. With respect to tumor size, colorectal SRCCs were classified into ≤4 cm, 4.1-6 cm, and >6 cm. The primary tumor location was divided into right colon, left colon, and rectum. Right colon consisted of appendix, cecum, ascending colon, hepatic flexure, and transverse colon; left colon consisted of splenic flexure, descending colon, and sigmoid colon, and rectum consisted of rectosigmoid junction and rectum [13]. According to clinical guidelines, a minimum of 12 lymph nodes should be examined for adequate staging and prognostic assessment in CRC [14]. Thus, the number of sampled lymph nodes was divided into <12 and ≥12. T stage and N stage were clearly classified based on the SEER registry. In terms of primary tumor treatment, we divided patients into local tumor excision, surgery, and no primary tumor treatment. For chemotherapy and radiation, patients were categorized into two groups, namely, yes and no/unknown.

### 2.3. Construction and Assessment of Nomogram

Patients were randomly assigned into the training cohort (*N* = 1466) and validation cohort (*N* = 584) by setting seed in the R software (training cohort: validation cohort = 7 : 3). Both univariate and multivariate logistic regression analyses were performed to identify independent predictors of LTSs. Afterwards, five independent risk factors (including race, marital status, T stage, N stage, and primary tumor treatment) were utilized to construct a nomogram model to predict LTS.

The calibration and discrimination capacities of the nomogram-based LTS prediction were assessed by a calibration plot in both training and validation cohorts. Besides, the C-index was also calculated. The receiving operator characteristic (ROC) curve and area under the curve (AUC) were further adopted and used to assess the predictive accuracy of the nomogram model.

### 2.4. Statistical Analysis

The chi-square test was used for comparison between LTSs and non-LTSs. Univariate logistic regression analysis was conducted to identify possible predictors of LTSs in nonmetastatic colorectal SRCC, and variables with *P* value < 0.05 in the univariate analysis were further analyzed by the multivariate model. Results were displayed as odds ratio (OR) with 95% confidence intervals (CIs). Kaplan-Meier method was used to plot survival curves, and log-rank test was employed to determine the statistical significance between groups. The SPSS statistics version 26.0 software (SPSS Inc., Chicago, United States) and R version 3.6.1 software (R Foundation for Statistical Computing, Vienna, Austria) were adopted for statistical analysis. A two-sided *P* value < 0.05 was considered as statistical significance.

## 3. Results

### 3.1. Baseline Characteristics of Patients

According to the inclusion and exclusion criteria (Figure 1), 2050 eligible patients were enrolled in our study, consisting of 1441 non-LTSs and 609 LTSs. When comparing the clinicopathological characteristics between non-LTSs and TLSs, we found that although age distribution was statistically significant between the two groups, there was no clear trend indicating the possible association between age and longer survival. Regarding race distribution, higher proportion of white patients was detected in the LTS group (86.37%) than that in the non-LTS group (80.01%). Sex proportion was not significantly different between the LTS group and non-LTS group (*P* = 0.148). With respect to marital status, there were significantly more married patients in the LTS group (60.92%) than those in the non-LTS group (51.77%). For tumor grade, not surprisingly, well differentiation and moderate differentiation accounted for a higher proportion in the LTS group than the non-LTS group. Tumor size ≤ 4 cm was relatively more common in the LTS group than the non-LTS group. For primary tumor location, patients of the LTS group had a higher proportion of right-sided colon cancer. There was no statistical difference of the number of sampled lymph nodes between the two groups. Regarding TNM stage, advanced T stage and N stage were definitely more common in the non-LTS group. In terms of tumor treatment, more patients underwent surgical resection or local tumor excision in the LTS group. However, relatively less patients received chemotherapy or radiation in the LTS group (Table 1).

### 3.2. Predictors of Long-Time Survivors in Nonmetastatic Colorectal SRCC

To identify predictors of LTSs in patients with nonmetastatic colorectal SRCC, patients were randomly assigned into the training and validation cohorts. Then, we performed univariate and multivariate logistic regression analyses to investigate independent predictors of LTSs. The results showed that white race (OR: 2.57, 95% CI: 1.60-4.30, *P* < 0.001), married status (OR: 1.34, 95% CI: 1.04-1.73, *P* = 0.023), less advanced tumor infiltration, negative lymph node involvement, and the performance of local tumor treatment (including radical surgery and local tumor excision) were independent predictors of being LTSs in patients with nonmetastatic colorectal SRCC (Table 2).

Interestingly, we found that marital status was an independent predictor. Thus, patients were divided into married and unmarried groups based on their marital status. We later performed stratified analysis to investigate the association between marital status and LTSs. As shown in Table 3, marital status was significantly associated with LTSs in the majority of subgroups.

### 3.3. Construction and Validation of Nomogram Model

**Figure 2:**
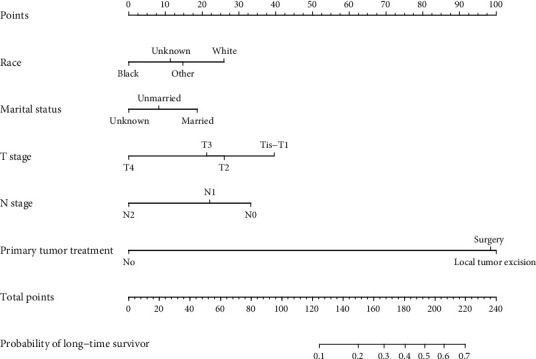
Nomogram to predict the probability of long-time survivor. For example, there was a black (0 point), married (20 points) patient with T1N1M0 (40 points for T1 and 22 points for N1) colorectal SRCC who received radical surgery (98 points). The five values summed to 180 points. For this specific patient, the probability of being a long-time survivor was approximately 0.4.

According to the multivariate logistic regression analysis, race, marital status, T stage, N stage, and primary tumor treatment were incorporated into a nomogram model to assign the probability of LTS in a specific individual. As shown in Figure 2, the performance of primary tumor treatment had the largest effect on the possibility of LTS, with a maximal score of 100. Other variables had varied effects on the probability of LTS.

**Figure 3:**
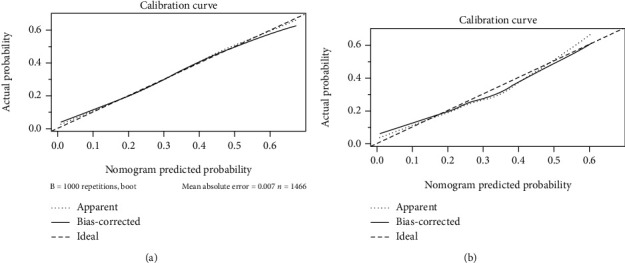
Calibration curve of nomogram-predicted and actual probability of long-time survivor in the training cohort ((a) *N* = 1466) and the validation cohort ((b) *N* = 584).

**Figure 4:**
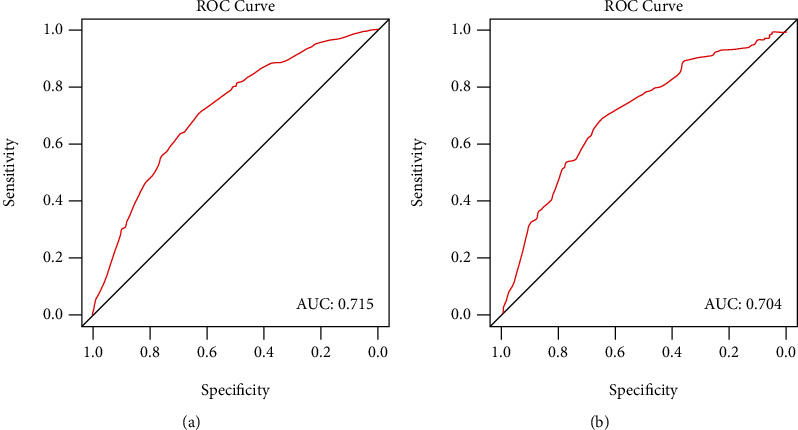
Receiver operating characteristic (ROC) curve and the under curve (AUCs) of ROC curves in the training cohort ((a) *N* = 1466) and the validation cohort ((b) *N* = 584).

The nomogram showed good accuracy in predicting LTS in the training cohort, with a C-index of 0.715 (Figure 3(a)). The calibration plot showed good agreement between the model predictions and actual observations for LTS (Figure 3(a)). Similarly, the C-index was 0.704 for the nomogram-based LTS prediction in the validation cohort (Figure 3(b)). As expected, the calibration curve showed good consistency of observed LTS probability with predicted LTS probability. Finally, ROC curve was adopted to assess the predictive power of the nomogram-based prediction model for LTS probability. As a result, the AUC was 0.715 and 0.704 in the training cohort and validation cohort, respectively (Figure 4).

## 4. Discussion

SRCC, a special histology of malignant tumors, is often found in the stomach and less common in other organs. Colorectal SRCC is a rare subtype of CRC, which consists of 0.1% to 2.6% of all CRC cases [6, 15]. Previous studies have revealed a female predominance in colorectal SRCC [16, 17], which is similar with that of gastric SRCC. Moreover, a younger age of onset has been reported in colorectal SRCC than differentiated colorectal adenocarcinoma [18, 19]. In terms of tumor location, several studies have reported that right colon is most commonly affected by colorectal SRCC [20, 21], because right-sided colon cancer has a higher incidence of microsatellite instability (MSI)-high, BRAF mutation, and CpG island methylation phenotype (CIMP)-high than that of left-sided colon cancer [22]. Thus, colorectal SRCC is a distinct entity compared to common colorectal adenocarcinoma. In consideration of the poor prognosis and aggressive tumor biology of colorectal SRCC, it is critical and intriguing to investigate the characteristics of patients who survive for a long time.

To the best of our knowledge, the present study was the first one to analyze the clinicopathological characteristics and to identify the independent predictors of LTSs in nonmetastatic colorectal SRCC. According to the multivariate logistic regression analysis, we found that white race, married status, less advanced T stage, negative lymph node metastasis, and primary tumor treatment (including radical surgery and local tumor excision) were significantly independent predictors of LTSs. Based on these results, we constructed a nomogram to predict LTSs, which is an easy-to-use and visual tool for clinical use. As shown in Figure 2, primary tumor treatment exerted the largest impact on the possibility of being LTSs, indicating the significant role of surgery in localized or locally advanced colorectal SRCC [23], especially in the era of multidisciplinary treatment of colorectal SRCC [24]. Other parameters (including race, marital status, T stage, and N stage) had relatively smaller effects. As a user-friendly statistical method, nomogram model could provide the possibility of being TLSs by formula calculation [25]. This nomogram-based model could assist clinicians to distinguish from high- and low-probability LTSs in nonmetastatic SRCC. For instance, when a black (0 point), married (20 points) patient with T1N1M0 (40 points for T1 and 22 points for N1) colorectal SRCC who received radical surgery (98 points), his possibility of surviving over 5 years is approximately 0.4 (180 points in total).

Intriguingly, we revealed that married patients with nonmetastatic colorectal SRCC were more likely to survive for a long time in the present study, indicating that marital status is a significantly prognostic factor. It is reasonable that spouse and family support plays a positive role in antitumor treatment and tumor surveillance [26]. Feng et al. have also revealed the similar findings [27], who suggest that the distress and psychological burden following tumor diagnosis could be shared and relieved by spouse support [28, 29]. Further stratified analyses of marital status and LTSs suggest that marital status is significantly associated with LTSs in most subgroups (Table 3). Consistently to most studies [18, 30], we revealed a proximal colon dominance for colorectal SRCC in our study (*N* = 1282, 62.5%).

Apart from these common and available clinicopathological factors analyzed above, recent studies have also revealed molecular factors that are associated with patient prognosis in nonmetastatic colorectal SRCC. Some authors suggest that colorectal SRCC may arise from a separate genetic pathway compared to common adenocarcinoma [31]. RAS/RAF/MAPK signaling is an important signaling pathway in the colorectal carcinogenesis. BRAF mutation is definitely associated with poor prognosis and resistance to the anti-EGFR treatment in CRC [32]. BRAF mutations have been reported to be common in SRCC, which can be as high as 20% [33]. In addition, BRAF mutation is significantly associated with CIMP positive status, with a relatively high incidence of MSI-H phenotype (24–48%) in colorectal SRCC [34, 35].

Colorectal SRCC is a distinct subtype of colorectal cancer. According to previous reports, colorectal SRCC presents as high-grade carcinoma and is more commonly associated with lymphatic invasion, vascular invasion, perineural invasion, and more advanced tumor stage [36, 37]. Besides, SRCC is a significantly prognostic factor for CRC [37]. In a recent nomogram predicting the overall survival of nonmetastatic colon cancer, SRCC is a significant predictor of poor prognosis [38]. Therefore, it is also intriguing to investigate the different predictors of LTSs between common colorectal adenocarcinoma and colorectal SRCC.

There are several limitations that should be discussed in our study. First, the intrinsic selection biases are unavoidable in this retrospective study. Second, the performance of chemotherapy and radiation is divided into two groups, namely, “yes” and “no/unknown.” However, we are unsure about the effects of radiochemotherapy on the long-time survival of patients, although our present results indicate negative impacts. Third, the model performance is overall acceptable in our study, both in the training cohort and validation cohort. However, external validation is still required to confirm the clinical application of our nomogram model.

## 5. Conclusion

To sum up, in this population-based study, we analyzed the clinicopathological characteristics of LTSs with nonmetastatic colorectal SRCC. Additionally, we also revealed several independent predictors of LTSs (including race, marital status, T stage, N stage, and primary tumor treatment) and further constructed a nomogram-based model for predicting the probability of LTSs, which showed acceptable performance in the training and validation cohorts.

## Figures and Tables

**Table 1:** Differences of clinicopathological characteristics between non-LTS^†^ and LTS among patients with nonmetastatic colorectal SRCC^‡^.

	Non-LTS			LTS	*P*
	≤2 years	2-5 years	Total		
Total	873	568	1441	609	
Age					0.04
≤40	68 (7.79)	63 (11.09)	131 (9.09)	46 (7.55)	
41-55	154 (17.64)	124 (21.83)	278 (19.29)	143 (23.48)	
56-70	253 (28.98)	179 (31.51)	432 (29.98)	197 (32.35)	
>70	398 (45.59)	202 (35.56)	600 (41.64)	223 (36.62)	
Race					0.003
Black	89 (10.19)	61 (10.74)	150 (10.41)	38 (6.24)	
White	698 (79.95)	455 (80.11)	1153 (80.01)	526 (86.37)	
Other	83 (9.51)	51 (8.98)	134 (9.3)	42 (6.9)	
Unknown	3 (0.34)	1 (0.18)	4 (0.28)	3 (0.49)	
Sex					0.148
Male	463 (53.04)	306 (53.87)	769 (53.37)	303 (49.75)	
Female	410 (46.96)	262 (46.13)	672 (46.63)	306 (50.25)	
Marital status					<0.001
Married	437 (50.06)	309 (54.4)	746 (51.77)	371 (60.92)	
Unmarried	395 (45.25)	234 (41.2)	629 (43.65)	218 (35.8)	
Unknown	41 (4.7)	25 (4.4)	66 (4.58)	20 (3.28)	
Tumor grade					<0.001
Well/moderately	37 (4.24)	48 (8.45)	85 (5.9)	62 (10.18)	
Poorly/undifferentiated	743 (85.11)	465 (81.87)	1208 (83.83)	469 (77.01)	
Unknown	93 (10.65)	55 (9.68)	148 (10.27)	78 (12.81)	
Tumor size (cm)					0.041
≤ 4	210 (24.05)	199 (35.04)	409 (28.38)	208 (34.15)	
4.1-6	261 (29.9)	148 (26.06)	409 (28.38)	148 (24.3)	
>6	281 (32.19)	171 (30.11)	452 (31.37)	177 (29.06)	
Unknown	121 (13.86)	50 (8.8)	171 (11.87)	76 (12.48)	
Tumor location					0.044
Right colon	527 (60.37)	351 (61.8)	878 (60.93)	404 (66.34)	
Left colon	120 (13.75)	90 (15.85)	210 (14.57)	90 (14.78)	
Rectum	209 (23.94)	118 (20.77)	327 (22.69)	105 (17.24)	
Unknown	17 (1.95)	9 (1.58)	26 (1.8)	10 (1.64)	
Number of lymph node examined					0.669
< 12	263 (30.13)	133 (23.42)	396 (27.48)	164 (26.93)	
≥ 12	597 (68.38)	430 (75.7)	1027 (71.27)	440 (72.25)	
Unknown	13 (1.49)	5 (0.88)	18 (1.25)	5 (0.82)	
T stage					<0.001
Tis-T1	43 (4.93)	34 (5.99)	77 (5.34)	65 (10.67)	
T2	23 (2.63)	45 (7.92)	68 (4.72)	51 (8.37)	
T3	433 (49.6)	306 (53.87)	739 (51.28)	390 (64.04)	
T4	374 (42.84)	183 (32.22)	557 (38.65)	103 (16.91)	
N stage					<0.001
N0	201 (23.02)	218 (38.38)	419 (29.08)	304 (49.92)	
N1	187 (21.42)	146 (25.7)	333 (23.11)	155 (25.45)	
N2	485 (55.56)	204 (35.92)	689 (47.81)	150 (24.63)	
Primary tumor treatment					<0.001
Surgery	778 (89.12)	551 (97.01)	1329 (92.23)	589 (96.72)	
Local tumor excision	12 (1.37)	6 (1.06)	18 (1.25)	14 (2.3)	
No	83 (9.51)	11 (1.94)	94 (6.52)	6 (0.99)	
Radiation					0.006
Yes	166 (19.01)	96 (16.9)	262 (18.18)	80 (13.14)	
No/unknown	707 (80.99)	472 (83.1)	1179 (81.82)	529 (86.86)	
Chemotherapy					0.04
Yes	468 (53.61)	329 (57.92)	797 (55.31)	306 (50.25)	
No/unknown	405 (46.39)	239 (42.08)	644 (44.69)	303 (49.75)	

^†^LTS: long-time survivor; ^‡^SRCC: signet ring cell carcinoma.

**Table 2:** Logistic regression analysis to identify predictors of long-time survivors in nonmetastatic colorectal SRCC^†^ in the training cohort.

Variables	Unadjusted logistic regression		Adjusted logistic regression	
	OR^‡^ (95% CI^**§**^)	*P*	OR (95% CI)	*P*
Age				
≤40	Reference		Reference	
41-55	1.69 (1.07-2.72)	0.028	1.29 (0.79-2.16)	0.320
56-70	1.35 (0.87-2.14)	0.186	0.79 (0.49-1.30)	0.338
>70	1.22 (0.79-1.91)	0.382	0.69 (0.42-1.15)	0.146
Race				
Black	Reference		Reference	
White	2.29 (1.47-3.69)	<0.001	2.57 (1.60-4.30)	<0.001
Other	1.37 (0.75-2.50)	0.305	1.84 (0.97-3.54)	0.064
Unknown	1.51 (0.07-12.41)	0.724	1.76 (0.08-15.05)	0.634
Sex				
Male	Reference			
Female	1.18 (0.95-1.47)	0.14		
Marital status				
Unmarried	Reference		Reference	
Married	1.42 (1.13-1.79)	0.003	1.34 (1.04-1.73)	0.023
Unknown	0.80 (0.42-1.43)	0.475	0.66 (0.33-1.25)	0.220
Tumor grade				
Well/moderately	Reference		Reference	
Poorly/undifferentiated	0.54 (0.36-0.80)	0.002	0.73 (0.48-1.12)	0.149
Unknown	0.80 (0.49-1.32)	0.380	0.98 (0.56-1.72)	0.951
Tumor size (cm)				
≤ 4	Reference			
4.1-6	0.79 (0.59-1.06)	0.117		
>6	0.79 (0.60-1.04)	0.099		
Unknown	0.74 (0.50-1.08)	0.126		
Tumor location				
Right colon	Reference		Reference	
Left colon	0.97 (0.70-1.32)	0.840	1.04 (0.72-1.47)	0.848
Rectum	0.69 (0.51-0.92)	0.012	0.91 (0.53-1.53)	0.719
Unknown	0.96 (0.39-2.19)	0.931	1.44 (0.56-3.44)	0.429
Number of lymph node examined				
< 12	Reference			
≥ 12	1.08 (0.84-1.39)	0.545		
Unknown	0.67 (0.19-1.90)	0.483		
T stage				
T4	Reference		Reference	
T3	2.43 (1.85-3.22)	<0.001	2.13 (1.59-2.88)	<0.001
T2	3.51 (2.15-5.70)	<0.001	2.42 (1.43-4.11)	0.001
Tis-T1	4.43 (2.79-7.06)	<0.001	3.64 (2.07-6.45)	<0.001
N stage				
N2	Reference		Reference	
N1	1.91 (1.41-2.59)	<0.001	2.07 (1.50-2.87)	<0.001
N0	3.35 (2.57-4.38)	<0.001	2.87 (2.09-3.96)	<0.001
Primary tumor treatment				
No	Reference		Reference	
Surgery	14.44 (4.49-88.28)	<0.001	21.38 (6.28-134.38)	<0.001
Local tumor excision	30.50 (6.63-223.18)	<0.001	22.80 (4.68-172.76)	<0.001
Radiation				
No/unknown	Reference		Reference	
Yes	0.64 (0.46-0.87)	0.006	0.70 (0.39-1.25)	0.234
Chemotherapy				
No/unknown	Reference		Reference	
Yes	0.70 (0.56-0.88)	0.002	1.00 (0.74-1.35)	0.984

^†^SRCC: signet ring cell carcinoma; ^‡^OR: odds ratio; ^**§**^CI: confidence interval.

**Table 3:** The association between marital status and long-time survivors in nonmetastatic colorectal SRCC^**†**^.

Variables	Married				Unmarried				*P* value
	Total		LTS^‡^		Total		LTS		
	*N*	%	*N*	%	*N*	%	*N*	%	
Total	1117		371		847		218		
Age									<0.001
≤40	77	6.89	28	7.55	91	10.74	16	7.34	
41-55	252	22.56	96	25.88	144	17	42	19.27	
56-70	394	35.27	135	36.39	215	25.38	58	26.61	
>70	394	35.27	112	30.19	397	46.87	102	46.79	
Race									<0.001
Black	72	6.45	13	3.5	105	12.4	23	10.55	
White	926	82.9	326	87.87	688	81.23	184	84.4	
Other	112	10.03	29	7.82	54	6.38	11	5.05	
Unknown	7	0.63	3	0.81	0	0	0	0	
Sex									<0.001
Male	686	61.41	224	60.38	347	40.97	71	32.57	
Female	431	38.59	147	39.62	500	59.03	147	67.43	
Tumor grade									0.096
Well/moderately	92	8.24	43	11.59	50	5.9	17	7.8	
Poorly/undifferentiated	898	80.39	281	75.74	709	83.71	175	80.28	
Unknown	127	11.37	47	12.67	88	10.39	26	11.93	
Tumor size (cm)									0.017
≤ 4	360	32.23	140	37.74	233	27.51	64	29.36	
4.1-6	309	27.66	90	24.26	231	27.27	55	25.23	
>6	309	27.66	93	25.07	287	33.88	75	34.4	
Unknown	139	12.44	48	12.94	96	11.33	24	11.01	
Tumor location									0.006
Right colon	665	59.53	235	63.34	559	66	152	69.72	
Left colon	182	16.29	56	15.09	103	12.16	32	14.68	
Rectum	254	22.74	74	19.95	167	19.72	30	13.76	
Unknown	16	1.43	6	1.62	18	2.13	4	1.83	
Number of lymph node examined									0.854
< 12	311	27.84	101	27.22	231	27.27	60	27.52	
≥ 12	793	70.99	266	71.7	608	71.78	157	72.02	
Unknown	13	1.16	4	1.08	8	0.94	1	0.46	
T stage									
Tis-T1	79	7.07	42	11.32	54	6.38	20	9.17	0.491
T2	63	5.64	33	8.89	54	6.38	17	7.8	
T3	627	56.13	233	62.8	453	53.48	145	66.51	
T4	348	31.15	63	16.98	286	33.77	36	16.51	
N stage									0.075
N0	403	36.08	185	49.87	294	34.71	107	49.08	
N1	244	21.84	90	24.26	222	26.21	61	27.98	
N2	470	42.08	96	25.88	331	39.08	50	22.94	
Primary tumor treatment									0.318
No	47	4.21	3	0.81	48	5.67	3	1.38	
Surgery	1051	94.09	361	97.3	786	92.8	208	95.41	
Local tumor excision	19	1.7	7	1.89	13	1.53	7	3.21	
Radiation									0.105
No/unknown	912	81.65	312	84.1	716	84.53	198	90.83	
Yes	205	18.35	59	15.9	131	15.47	20	9.17	
Chemotherapy									<0.001
No/unknown	473	42.35	171	46.09	437	51.59	120	55.05	
Yes	644	57.65	200	53.91	410	48.41	98	44.95	

^†^SRCC: signet ring cell carcinoma; ^‡^LTS: long-time survivor.

## Data Availability

Data are available from the corresponding author upon reasonable request.
